# Assessment of Postnatal Pulmonary Adaption in Bovine Neonates Using Electric Impedance Tomography (EIT)

**DOI:** 10.3390/ani11113216

**Published:** 2021-11-10

**Authors:** Ulrich Bleul, Corina Wey, Carolina Meira, Andreas Waldmann, Martina Mosing

**Affiliations:** 1 Department of Farm Animals, Clinic of Reproductive Medicine, Vetsuisse-Faculty University Zurich, 8057 Zurich, Switzerland; corina.wey@gmx.ch; 2 Department of Clinical Diagnostics and Services, Section Anaesthesiology, Vetsuisse Faculty, University of Zurich, 8057 Zürich, Switzerland; cmeira@vetclinics.uzh.ch; 3 Department of Anesthesiology and Intensive Care Medicine, Rostock University Medical Center, 39071 Rostock, Germany; awa091086@gmail.com; 4 Department of Veterinary Anaesthesia and Analgesia, School of Veterinary Medicine, College of Science, Health, Engineering and Education, Murdoch University, Murdoch 6150, Australia; M.Mosing@murdoch.edu.au

**Keywords:** neonate, cattle, lung ventilation, tidal impedance change, EIT, blood gas parameter

## Abstract

**Simple Summary:**

The lungs of the neonate must oxygenate the blood and eliminate carbon dioxide at birth. Efficient gas exchange requires ventilation of the pulmonary alveoli to meet rapidly increasing oxygen demands. It is not entirely clear how quickly the different regions of the lungs become ventilated and when ventilation of all lung regions is achieved. We used electric impedance tomography (EIT) to measure lung stretch and aeration in 20 non-sedated neonatal calves born without difficulty. For EIT measurement, an electrode belt was placed around the thorax of each calf positioned in sternal recumbency immediately after birth and at various other times until 3 weeks of age to measure aeration and ventilation in different regions of the lungs. Standard EIT variables describing distribution of ventilation, respiratory rate, and several blood gas variables were determined. Most lung regions were ventilated immediately after birth. Ventilation of the ventral regions increased during the first hour, after which it decreased. Dorsal lung regions and the right lung had the greatest ventilation. The respiratory rate decreased, and the tidal impedance change as a surrogate for tidal volume increased in the first 3 weeks of life. The study showed that most regions of the lungs are ventilated immediately after birth. However, this is followed by a reduction in ventilation of ventral regions of the lungs, presumably because of gravity-driven ventral movement of residual amniotic fluid. These findings may allow identification of calves with insufficient lung expansion and ventilation after birth.

**Abstract:**

Several aspects of postnatal pulmonary adaption in the bovine neonate remain unclear, particularly the dynamics and regional ventilation of the lungs. We used electric impedance tomography (EIT) to measure changes in ventilation in the first 3 weeks of life in 20 non-sedated neonatal calves born without difficulty in sternal recumbency. Arterial blood gas variables were determined in the first 24 h after birth. Immediately after birth, dorsal parts of the lungs had 4.53% ± 2.82% nondependent silent spaces (NSS), and ventral parts had 5.23% ± 2.66% dependent silent spaces (DSS). The latter increased in the first hour, presumably because of gravity-driven ventral movement of residual amniotic fluid. The remaining lung regions had good ventilation immediately after birth, and the percentage of lung regions with high ventilation increased significantly during the study period. The centre of ventilation was always dorsal to and on the right of the theoretical centre of ventilation. The right lung was responsible for a significantly larger proportion of ventilation (63.84% ± 12.74%, *p* < 0.00001) compared with the left lung. In the right lung, the centrodorsal lung area was the most ventilated, whereas, in the left lung, it was the centroventral area. Tidal impedance changes, serving as a surrogate for tidal volume, increased in the first 3 weeks of life (*p* < 0.00001). This study shows the dynamic changes in lung ventilation in the bovine neonate according to EIT measurements. The findings form a basis for the recognition of structural and functional lung disorders in neonatal calves.

## 1. Introduction

To establish postnatal lung ventilation, it is essential that the fluids in the lungs are initially removed. During parturition, amniotic fluid in the foetal lungs is partially expelled by compression of the thorax, and the remainder is resorbed by the lungs and to a lesser extent expelled when respiration starts. Ventilation of the lungs is considered the most important mechanism for reabsorption of amniotic fluid; the hydrostatic pressure that occurs during inspiration facilitates movement of the fluid into the tissue [1]. Intranatal hyperkapnia and hypoxia and the resulting acidosis stimulate the respiratory centres in the pons and medulla. The surface tension of the uninflated alveoli, the elasticity of the lungs, and the viscosity of the residual amniotic fluid must be overcome during the first breath, which results in a pressure that is 6–20 times that of subsequent breaths [2]. More lung regions become progressively ventilated over time postnatally [3,4,5,6]. In studies of the new-born calf, the effect of increasing ventilation could so far only be indirectly reconstructed by means of pulse oximetry or arterial blood gas analyses [7,8,9]. However, using radiographic observations, early studies on babies showed that complete pulmonary ventilation is achieved after a few breaths to a few hours after birth [6,10,11]. Serial sonographic and radiographic examinations of the lungs showed that 64% of calves born without difficulty had ventilation of all lung regions as early as 1 h after birth [12]. Complete pulmonary ventilation seen via radiography and ultrasonography took up to 12 h in all calves. Areas with persistent incomplete ventilation appeared as radiodensities in the perihilar lung regions. In neonatal calves born via Caesarean section, monitoring of pulmonary ventilation via radiography showed that ventilation was not complete until 24 h postnatum, and fluid resorption occurred more quickly in the ventral rather than the dorsal areas of the lungs [13]. Computed tomography (CT) of the lungs of sedated calves revealed that the greatest increase in pulmonary ventilation occurred in the first 6 h postnatum [14]. In addition, the dorsal lung regions became completely ventilated before the ventral areas, and complete pulmonary ventilation did not occur until 14 days postnatum [14]. The varying results may have been attributable to differences in imaging modalities because CT and radiography were done in sedated or restrained calves, and ultrasonography only assessed certain regions of the lungs. Furthermore, imaging modalities provide static images of the lungs and cannot capture dynamic pulmonary changes.

Electric impedance tomography (EIT) provides a completely different approach to the assessment of pulmonary ventilation. It is a non-invasive and radiation-free imaging modality for the measurement of thoracic impedance changes in humans and different animal species [15,16,17]. Electric impedance decreases with increasing intrapulmonary gas volume and increases with increasing volume of blood or other fluids or when disruption of cellular barriers occurs [15]. An electrode belt with multiple electrodes is placed around the thorax of the human or animal patient [18]. Small alternating electrical currents are applied through pairs of electrodes, and the resulting voltages (surface potentials) are measured via the remaining electrodes. An alternating current is then applied sequentially to other electrode pairs until the entire thorax has been circumnavigated. Data from one electrode rotation are used to create an image, which is called a frame, and completion of one frame is followed by the start of another. Up to 50 frames are created per second allowing observation of the distribution and activity of ventilation over time [15,19]. The lung area reflected by an EIT image comprises a two-dimensional lentiform cross-section of the lungs because of the distribution of the current in the thorax [15,20].

The goal of this study was to describe the dynamic changes in pulmonary ventilation using EIT in calves from birth to 21 days postnatum. Endpoints were changes in the distribution of ventilation in the lungs and the time to obtain ventilation of all lung regions. A secondary goal was to investigate whether changes in pulmonary ventilation in the first 24 h postnatum are reflected in changes in blood gas variables.

## 2. Materials and Methods

### 2.1. Animals

A total of 20 calves born to three primiparous and 17 pluriparous cows from a university research herd were used. The calves were born after a mean gestation length of 289.60 ± 6.72 days and were delivered with light manual traction in five cows and without assistance in the remainder. Breeds of calves included Simmental (*n* = 7), Braunvieh × Simmental cross (*n* = 4), Braunvieh (*n* = 4), Simmental × Rotfleck cross (*n* = 2), and Braunvieh × Rotfleck cross (*n* = 3). There were nine heifer calves and 11 bull calves. A modified APGAR score test was done in each calf immediately postnatum to assess vitality [21]. This included the assessment of respiration, colour of the mucous membranes, reflex irritability, and spontaneous movement or muscle tone. Each criterion was scored from 0 to 2 with a maximum overall score of 8. Physical examination and evaluation of maturity were carried out in all calves [22]. The calves received 5 L of colostrum from their dams within the first 12 h of life. Body weight was determined before the first colostrum feeding and after the last EIT measurement, 504 h postnatum.

### 2.2. Measurements Using Electric Impedance Tomography

**Figure 1:**
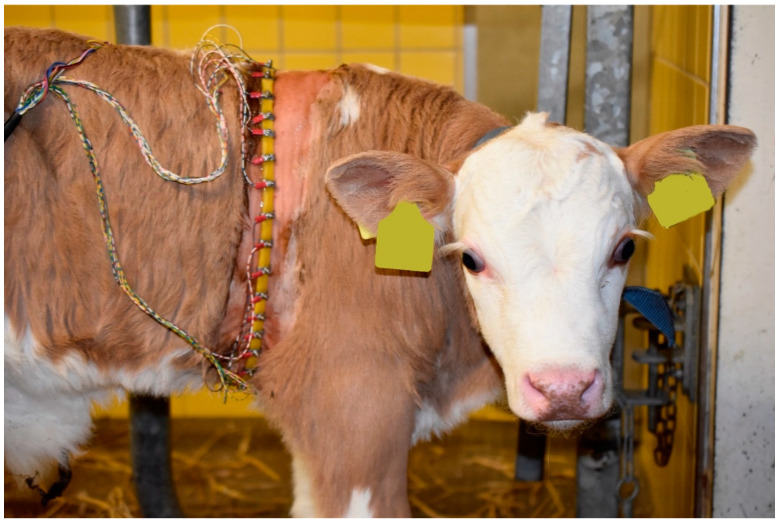
One week old calf with a rubber belt with 32 evenly distributed electrodes for electrical impedance tomography. The belt is placed at the level of the sixth intercostal space.

Electrical impedance tomography was done using the BB-Vet (SenTec, Landquart, Switzerland), which contains a monitor and measuring unit connected to a belt with 32 equally mounted electrodes. Depending on the size of the calf, one of three rubber belt lengths was used: 58.5 cm, 75.5 cm, or 77.5 cm (in the non-stretched state). The belt was placed around the thorax so that it crossed the sixth intercostal space at midlevel (Figure 1).

**Figure 2:**
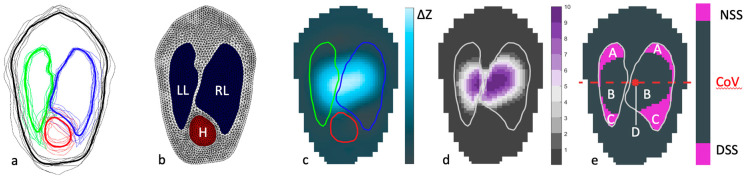
(**a**–**c**) Construction of a finite element model: (**a**) lung, heart, and thorax contour based on eight segmented CT scans (thin lines denote single animals, thick line mean contours); (**b**) finite element model based on mean contour (LL: left lung, RL: right lung, H: heart); (**c**) ventilation distribution based on the ventilation-induced impedance changes; (**d**) electrical impedance tomogram showing pixel grid and colour coding of ventilated lung regions. Colour bar showing the impedance changes divided into 10 categories of relative tidal stretch. (**e**) Electrical impedance tomogram showing the centre of ventilation and the silent spaces. A: nondependent silent spaces (NSS); B: ventilated lung regions; C: dependent silent spaces (DSS); D: centre of ventilation (CoV) with ventilation horizon.

Before the start of the study, a finite element model was created in order to display the results of the EIT measurements at the anatomically correct locations [23,24]. The finite element model was obtained through segmentation of a computed tomography scan image (Figure 2a–c) in which the electrode plane was identified (sixth intercostal space) in six neonatal calves from a previous study [25,26]. This plane was chosen because it corresponds to 50% of the length of the thorax, covering a large lung area, there is no interference with abdominal organs, and landmarks for placement of the electrode belt are straightforward. In the corresponding DICOM file, the heart, the lungs, and the area of the thorax were segmented using the ITK-SNAP software (retrieved 3 January 2016, from http://www.itksnap.org) and exported as vtk-files. Matlab (MathWorks, Natick, MA, USA) was used to align and to calculate an average contour from all calves and to generate the corresponding average finite element model (Figure 2b) [17].

Electrical impedance tomography was done using a current amplitude of 3 mA and a frame rate of 50 frames per second at 0, 15, 30, and 45 min and 1, 2, 4, 6, 8, 12, 24, 48, 72, 96, 120, 144, 168, 216, 264, 312, 360, 408, 456, and 504 h postnatum. After the first four measurements, the hair over the sixth intercostal space was clipped on both sides of the thorax circumferentially to ensure consistent positioning of the belt and improve contact between electrodes and skin. To further enhance electrode contact, a gel layer (Vetogel, Streuli, Uznach, Switzerland) was applied between skin and electrode belt before each measurement. Individual measurements included at least 21 respiratory cycles. Except for the first hour when frequent measurements were made, the belt was applied at least 10 min before the start of measurements to accustom the calf to the device. 

The raw EIT data were analysed retrospectively using ibeX software (SenTec, Landquart, Switzerland), and the variables centre of ventilation, dependent (DSS) and nondependent (NSS) silent spaces, stretch, regional ventilation in the right and left lung, respiratory rate, and tidal impedance change as a surrogate for tidal volume (ΔZVT) were calculated. The data were transferred to an Excel (Microsoft Windows, Wallisellen, Switzerland) data sheet, and the tidal impedance change per minute was calculated from the respiratory rate and tidal impedance change.

#### 2.2.1. Centre of Ventilation

The centre of ventilation (CoV) describes laterolateral and ventrodorsal shifts in the distribution of lung ventilation and has been defined as the weighted mean of the geometrical centres of ventilation [27,28]. The CoV is calculated along the *x*-axis from right to left (CoV_rl_) and along the *y*-axis from ventral to dorsal (CoV_vd_) as follows [27,29]:

(1) $${\mathrm{CoV}}_{\mathrm{rl}}=\frac{{{}^{\sum }}_{\left\{x,\;y\right\}\in lung^{x\cdot TI_{\left[x,y\right]}}}}{{{}^{\sum }}_{\left\{x,\;y\right\}\in lung^{TI_{\left[x,y\right]}}}},$$

(2) $${\mathrm{CoV}}_{\mathrm{vd}}=\frac{{{}^{\sum }}_{\left\{x,\;y\right\}\in lung^{y\cdot TI_{\left[x,y\right]}}}}{{{}^{\sum }}_{\left\{x,\;y\right\}\in lung^{TI_{\left[x,y\right]}}}},$$

where *x* is the horizontal distance of each pixel from 0 positioned rightmost, *y* is the vertical distance of each pixel from 0 positioned ventral-most, and *TI* is the tidal image, representing tidal impedance changes of all pixels within the lung area.

The CoV is expressed as percentages from right to left and from ventral to dorsal. CoV_rl_ = 0% indicates that the centre is located rightmost, and CoV_rl_ = 100% describes a leftmost position. The description of the CoV in the vertical axis is analogous; CoV_vd_ = 0% and 100% indicates ventral-most and dorsal-most, respectively (Figure 2e). The theoretical centre of ventilation of a uniformly inflated lung is defined as the intersection of the two lines in the middle of the horizontal and vertical lung diameters.

#### 2.2.2. Relative Tidal Stretch and Silent Spaces

Relative tidal stretch and silent spaces are variables that provide information about changes in the lungs that are related to inspiration and expiration. The relative tidal stretch describes impedance changes in certain regions of the lungs during one breath compared with other regions of the lungs [30]. For calculation of the tidal stretch, a 32 × 32 pixel impedance distribution map was applied to the lung area. The impedance was measured at each pixel at the beginning and at the end of inhalation, and then the impedance changes were calculated. The differences in impedance were divided into 10 categories, and each pixel was assigned to a category. The transition from the category with the smallest differences to the category with the greatest differences followed a colour pattern that ranged from dark grey to light grey to light purple to dark purple (Figure 2d). Higher categories (in the upper part of the colour bar with darker shades of purple) had greater impedance changes and, therefore, more pulmonary ventilation, while the opposite is true for the categories in the lower part of the colour bar (dark grey), which were characterised by less intense pulmonary ventilation.

In the lung-function view, the pixels with the lowest impedance changes, which represented minimally or nonventilated lung regions, were highlighted in pink (Figure 2e). The ventilation horizon and the horizontal reference line across the CoV were also calculated. In standing calves or calves in sternal recumbency, the silent spaces located above the ventilation horizon were defined as nondependent silent spaces, which were not gravity-dependent. The silent spaces below the ventilation horizon were calculated and expressed in the same fashion and defined as dependent silent spaces, which represented areas dependent on gravity [29]. The NSS and DSS were expressed as a percentage of the total number of pixels.

#### 2.2.3. Global and Regional Ventilation of the Right and Left Lung

Impedance change was measured within each pixel of the lung area at the start and at the end of inhalation. On the basis of the impedance distribution, the ventilation of the left and right lung was evaluated. Each lung was further divided into four regions: right and left ventral (ΔZVT_v_), centroventral (ΔZVT_cv_), centrodorsal (ΔZVT_cd_), and dorsal (ΔZVT_d_) region. 

#### 2.2.4. Respiration Rate, Tidal Impedance Change, and Minute Tidal Impedance Change

Determination of respiratory rate (RR) was based on the maximum impedance changes during a respiratory cycle, and the tidal impedance change (ΔZVT) of all pixels was calculated and used as a surrogate for tidal volume. The ΔZVT is expressed in arbitrary units (AU), which is a relative measure [31]. The minute tidal impedance change (ΔZVTmin) was calculated as a function of respiratory rate and ΔZVT, expressed in AU/min.

### 2.3. Blood Gas Analysis

Venous blood was collected from a jugular vein immediately after birth using an 18 G hypodermic needle (Sterican, B. Braun, Melsungen, Germany) and a lithium heparin blood gas syringe (Monovette, Sarstedt, Nümbrecht-Rommelsdorf, Germany), and subsequent samples were collected from the medial intermediate branch of the caudal auricular artery 15 min and 2, 6, 12, and 24 h after birth [7,32]. After the hair over the artery was clipped and the area was cleaned and disinfected, a 24 G needle (Henke-Ject, Henke-Sass, Wolf, Tuttlingen, Germany) was used to puncture the artery and blood was collected into a lithium heparin capillary tube (Blood Gas blood collection capillary tube; Siemens Healthcare Diagnostics, Camberley, UK). Venous and arterial samples were analysed immediately after collection using the RapidPoint 500 blood gas analyser (Siemens Healthcare Diagnostics, Camberley, UK). The pH, temperature-corrected partial pressure of carbon dioxide (pCO_2_) and oxygen (pO_2_), oxygen saturation (sO_2_), and base excess were determined.

The variables described below were calculated on the basis of blood gas results and with the help of barometric data from MeteoSchweiz [33].

#### 2.3.1. Alveolar–Arterial Oxygen Difference

To calculate the alveolar–arterial oxygen difference (A–a gradient), the alveolar partial pressure of oxygen pAO_2_ was first calculated as follows:

pAO_2_ = FiO_2_ (pAtm − pH_2_O) − (paCO_2_/RQ), (3)

where FiO_2_ is the inspired fraction of oxygen from natural air set at 0.21, pAtm is the prevailing barometric pressure (mmHg), pH_2_O is partial water vapour pressure (47 mmHg), paCO_2_ is the arterial partial pressure of carbon dioxide (mmHg), and RQ is the respiratory quotient (1).

A–a gradient = pAO_2_ − paO_2_, (4)

where pAO_2_ is the alveolar partial pressure of oxygen (mmHg), and paO_2_ is the arterial partial pressure of oxygen (mmHg).

#### 2.3.2. Oxygen Content-Based Index (Fshunt)

To calculate the Fshunt, the pulmonary end-capillary oxygen content (Cc’O_2_ (5)) and the arterial oxygen content CaO_2_ (6) were calculated.

Cc’O_2_ (mL O_2_/dL blood) = tHb × 1.39 × Sc’O_2_ + 0.003 × pAO_2_, (5)

where tHb is the total haemoglobin (g/dL), 1.39 is the oxygen-carrying capacity of haemoglobin (mL/g) [34], Sc’O_2_ is the oxygen saturation of the pulmonary end-capillary (1 = 100%) [35], and 0.003 is the solubility coefficient of oxygen in plasma (mL·100 mL^−1^·mmHg^−1^) [34,36].

CaO_2_ (mL O_2_/dL blood) = (tHb × 1.39 × sO_2_) + (0.003 × paO_2_), (6)

where sO_2_ is the arterial oxygen saturation.

Fshunt = ((Cc’O_2_ − CaO_2_)/(Cc’O_2_ − CaO_2_ + 3.5)) × 100, (7)

where 3.5 is estimate of the mixed arteriovenous oxygen content (mL/dL) [37].

### 2.4. Statistical Analysis

Using the program StatEL (AdScience, Paris, France), means and standard deviations were calculated for continuous variables, and the data were tested for normality using the Shapiro–Wilks W-test. The differences in body weight were analysed using the *t*-test. Linear correlations were examined using Pearson correlation, and multiple regression analysis was used for analysis of the relationship between multiple variables. Paired samples or repeated measures were analysed using the Friedman test or repeated-measures ANOVA, and a post hoc test (Nemenyi Test or Fisher LSD Test with Bonferroni correction) was applied when differences were significant. With respect to the regional ventilation, ventilation of the lungs was compared using the Wilcoxon test. Differences were considered significant at *p* < 0.05.

## 3. Results

### 3.1. Clinical Variables

None of the calves had signs of asphyxia after birth or of respiratory disease during the study period. The APGAR scores ranged from 4–8 (mean ± standard deviation, 6.95 ± 0.83). The mean birth weight was 48.75 ± 7.38 kg, and the weight increased to 63.18 ± 9.55 kg (*p* < 0.0001) by the end of the study; birth weights and final weights were closely correlated (*r* = 0.94, *p* < 0.0001).

### 3.2. Centre of Ventilation

**Figure 3:**
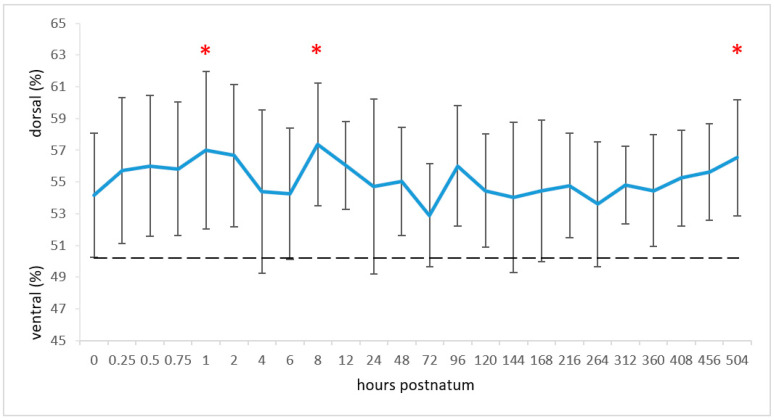
Means and standard deviations of the centre of ventilation (CoV; blue line) in the ventrodorsal lung diameter and CoV for equal distribution between the ventral and dorsal regions (black dashed line). The measuring times are graphed equidistantly. Values with an asterisk differ from the value at time 0.

The mean CoV_rl_ during the entire study period was 48.13% ± 6.13% and, thus, the centre was slightly to the right of the theoretical centre of ventilation. The CoV_vd_ was in the dorsal region of the lungs throughout the study period with a mean of 55.16% ± 4.05%. The CoV_vd_ underwent significant changes during the study (*p* < 0.01; Figure 3). Within the first hour, there was a significant change in the dorsal direction from 54.16% ± 3.90% to 57.00% ± 4.96% (*p* < 0.01). Eight hours later, the mean position was at 57.37% ± 3.88%, which was the maximum value for the entire study period. This value differed significantly from the values at times 0 and 6 h (both *p* < 0.01). On day 3, the CoV_vd_ had a minimum value of 52.90% ± 3.26%, which was significantly different from the value at 8 h (*p* < 0.001). This was followed by a dorsal shift of the CoV_vd_, which resulted in a significant difference between the first (birth) and the last measurement (end of study) (*p* < 0.05).

### 3.3. Relative Tidal Stretch and Silent Spaces

Significant changes in relative tidal stretch occurred over time in categories 1, 5, 8, 9, and 10. The relative tidal stretch values of category 5 decreased over time (*p* < 0.01); the values at times 4 h and days 3, 7, 9, 11, 13, and 19 were significantly lower than at time 0 (all *p* < 0.05). The values of categories 8 and 9 increased significantly over time (both *p* < 0.001). The percentage of category 8 increased from 13.07% ± 1.43% to 14.91% ± 2.49%, and the percentage of category 9 increased from 12.96% ± 1.89% to 14.94% ± 3.34%. The values of category 10 also changed over time (*p* < 0.05) but only the value on day 3 exceeded that of time 0 (*p* < 0.05).

**Figure 4:**
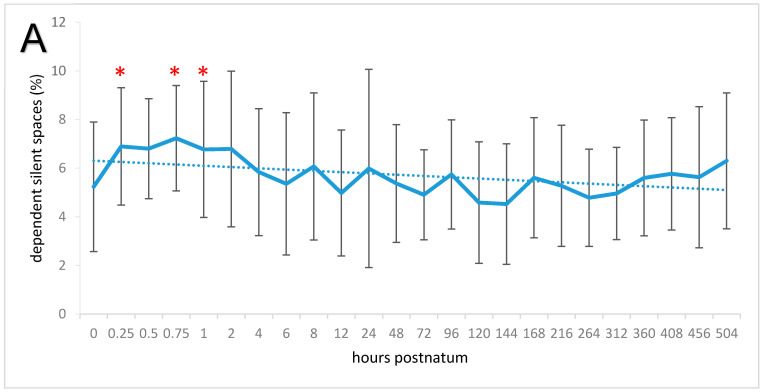

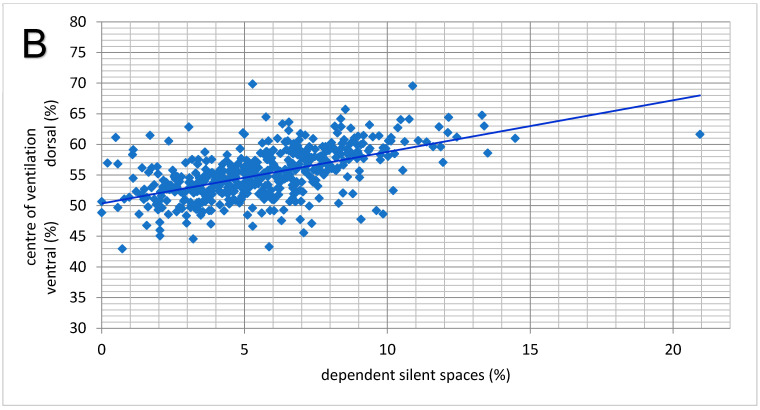
(**A**) Means and standard deviations of the dependent silent spaces (poorly or nonventilated ventral lung regions; solid line) and trend line (dotted) in 20 neonatal calves. The measuring times are graphed equidistantly. Values with an asterisk differ from the value at time 0. (**B**) Correlation between percentage values of the ventrodorsal centre of ventilation and the dependent silent spaces (*r* = 0.55; *p* < 0.00001).

The mean amount of category 1 representing silent spaces decreased during the study period (*p* < 0.05), and lower values were measured on days 3 and 13 compared with time 0 (*p* < 0.05). The proportion of NSS area was 4.53% ± 2.82% immediately after birth, and the mean for the entire study period was 3.66% ± 3.04%. The overall proportion of DSS was 5.71% ± 2.64%, and there were significant changes over time (*p* < 0.01; Figure 4A). The proportion increased in the first hour from 5.23% ± 2.66% to 6.77% ± 2.80% (*p* < 0.05); the maximum of 7.23% ± 2.17% was measured 45 min after birth. The DSS subsequently decreased and reached a minimum of 4.52% ± 2.48% on day 6. This was different from the values at 45 min and 1 h (both *p* < 0.001). 

DSS and the CoV_vd_ had a strong positive correlation (*r* = 0.55; *p* < 0.00001; Figure 4B). Furthermore, immediately after birth the APGAR score had a significant and negative correlation with the DSS (*r* = −0.56; *p* < 0.05). 

### 3.4. Regional Ventilation of the Right and Left Lung

**Figure 5:**
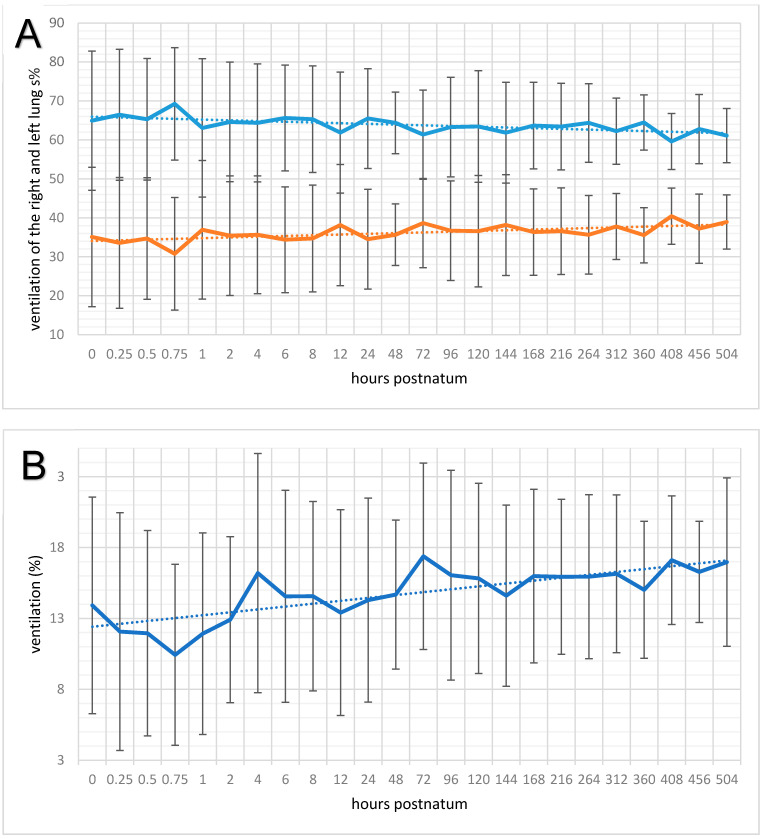
(**A**) Means and standard deviations of ventilation of the right (blue solid line) and left lungs (orange solid line) and trend line (dotted) in 20 neonatal calves. The measuring times are graphed equidistantly. (**B**) Means and standard deviations of the ventilation of the centrovental lung region of the left lung (solid line) and trend line (dotted) in 20 neonatal calves. The measuring times are graphed equidistantly.

On average, 63.84% ± 12.74% of the ΔZVT was in the right lung (Figure 5A); 5.66% ± 4.30% of the ventilation was in the ventral, 19.80% ± 7.00% was in the centroventral, 25.76% ± 6.20% was in the centrodorsal, and 12.62% ± 7.62% was in the dorsal region. Thus, of the inhaled air, almost two-thirds entered the right lung and slightly more than one-third entered the left lung (*p* < 0.00001). In the left lung, 3.07% ± 2.34% of all inhaled air was seen ventrally, 14.76% ± 6.61% was seen centroventrally, 14.05% ± 6.52% was seen centrodorsally, and 4.29% ± 4.07% was seen dorsally. This distribution did not change significantly during the study period. Among all eight lung regions, there were significant changes in the ventilated areas over time in the left ΔZVT_cv_ (*p* < 0.01); it increased from 13.92% ± 7.63% at birth to 16.97% ± 5.94% at 21 days (Figure 5B).

### 3.5. Respiration Rate, Global Ventilation (ΔZVT), and Minute Tidal Impedance Change

**Figure 6:**
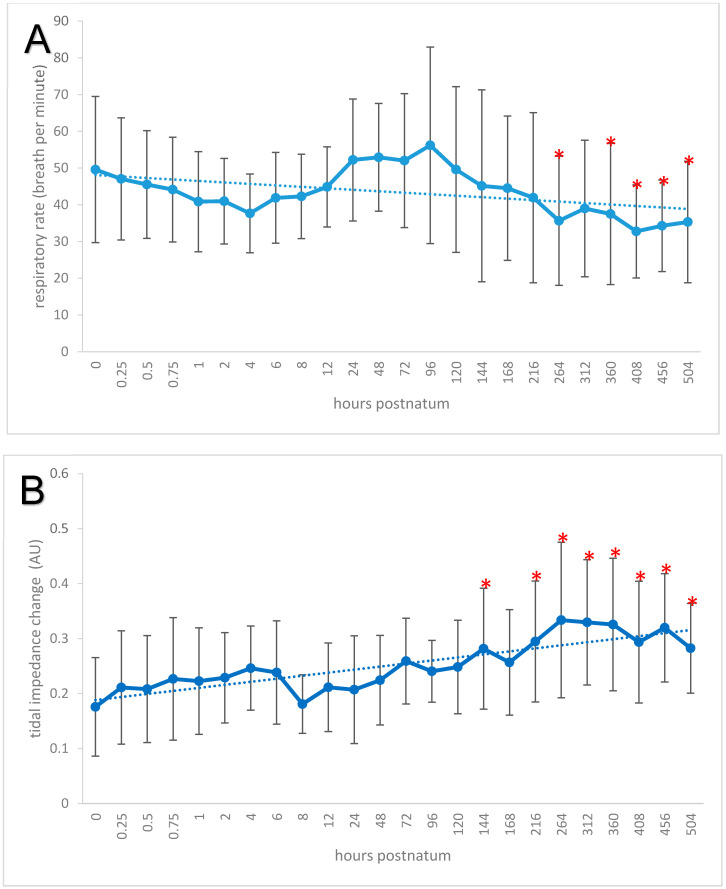

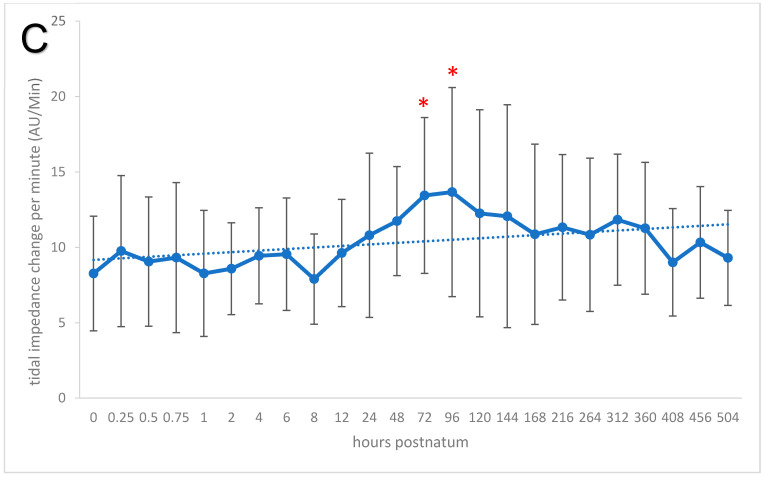
(**A**) Means and standard deviations of the respiratory rate (solid line) and trend line (dotted) in 20 neonatal calves. The measuring times are graphed equidistantly. Values with an asterisk differ from the value at time 0. (**B**) Means and standard deviations of the tidal impedance change (solid line) and trend line (dotted) in 20 neonatal calves. The measuring times are graphed equidistantly. Values with an asterisk differ from the value at time 0. (**C**) Means and standard deviations of the minute tidal impedance change (solid line) and trend line (dotted) in 20 neonatal calves. The measuring times are graphed equidistantly. Values with an asterisk differ from the value at time 0.

The respiratory rate decreased significantly from 49.58 ± 19.90 breaths per minute at birth to 35.31 ± 16.53 breaths per minute at 21 days (*p* < 0.00001; Figure 6A). A minimum of 37.66 ± 10.71 breaths per minute was reached 24 h after birth (post hoc test *p* = 0.0078). From day 11 onwards, the mean respiratory rate remained significantly lower than immediately after birth. Multiple regression with the dependent blood gas variables pH, paO_2_, and paCO_2_ showed a weak negative correlation between respiratory rate and pH (*r* = 0.14; *p* < 0.05).

Mean ΔZVT was 0.25 ± 0.09 AU, and significant increases were seen during the study period (*p* < 0.00001; Figure 6B). It increased from 0.18 ± 0.09 AU at birth to 0.28 ± 0.11 AU on day 6 (*p* < 0.001), and, from day 9 onwards, all values were significantly higher than at birth. The maximum value was 0.33 ± 0.11 AU on day 11, and the last measurement on day 21 was 0.28 ± 0.08 AU. In multiple regression, the value of ΔZVT was determined by pH, paO_2_, and paCO_2_ (V_ΔZ_ = 0.24 × pH + 0.00003 × paCO_2_ + 0.0017 × paO_2_ − 1.81; *p* < 0.05); paO_2_ was the only factor that was weakly correlated with ΔZVT (*r* = 0.24; *p* < 0.05), but ΔZVT was not correlated with body weight. The ΔZmin on days 3 (13.44 ± 5.17 AU/min) and 4 (13.66 ± 6.93 AU/min) were significantly higher than 8.27 ± 3.81 AU/min at birth (*p* < 0.01; Figure 6C).

### 3.6. Blood Gas Analysis

During the first day of life, pH increased on average from 7.21 ± 0.08 to 7.41 ± 0.03 (*p* < 0.00001), base excess increased from −2.37 ± 4.64 mmol/L to 3.76 ± 2.40 mmol/L (*p* < 0.00001), paO_2_ increased from 51.93 ± 12.54 to 71.49 ± 12.50 mmHg (*p* < 0.01), and paCO_2_ decreased from 55.29 ± 4.01 to 47.15 ± 4.47 mmHg (Table 1; *p* < 0.001). There was a weak positive correlation between ΔZVT and paO_2_ (*r* = 0.23, *p* = 0.034), and a weak negative correlation between the DSS of the ventral lung regions and the paO_2_ (*r* = −0.20, *p* = 0.025).

Parallel to the paO_2_ (*p* < 0.001), the arterial sO_2_ increased gradually from 85.93% ± 7.71% at 15 min to 94.93% ± 2.09% at the last measurement.

The mean pAO_2_ was 90.23 ± 7.24 mmHg. It increased from 85.31 ± 4.22 to 93.40 ± 4.66 mmHg in the first 24 h (*p* < 0.00001), and a maximum of 95.20 ± 8.53 mmHg was measured at 12 h.

The mean A–a gradient was 24.94 ± 12.08 mmHg. This variable changed significantly over time (*p* < 0.001) and decreased from 33.38 ± 10.54 mmHg at 15 min to 23.37 ± 9.76 mmHg at 2 h (Figure 6). The A–a gradient did not correlate with EIT variables.

**Figure 7:**
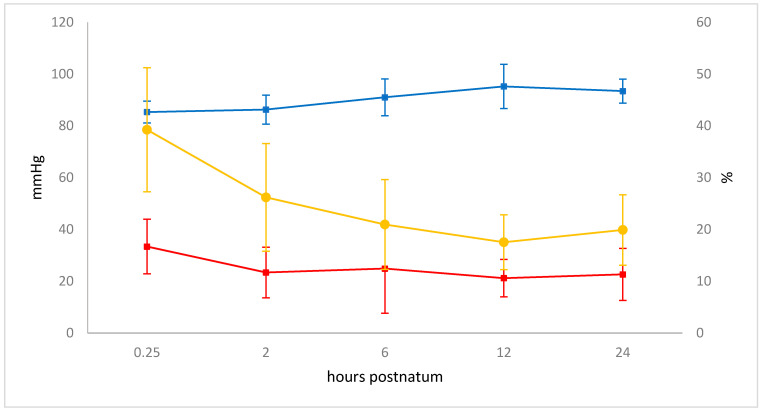
Changes in pAO_2_ (blue, *p* < 0.00001), A–a gradient (red, *p* < 0.001), and Fshunt (yellow, *p* < 0.00001) including standard deviations during the study period. The measuring times are graphed equidistantly.

The mean Fshunt was 24.67% ± 11.86%. It decreased in the first 24 h from 39.24% ± 11.96% to 19.90% ± 6.80% (*p* < 0.00001; Figure 7) and was also not correlated with EIT variables.

## 4. Discussion

Neonatal calves undergo drastic physiological changes immediately after birth. The organ of gas exchange must rapidly change from placenta to lungs; thus, a functional respiratory centre and rapid and efficient lung aeration are prerequisites for their survival. Adaption of the lungs to extrauterine life has been a research focus in various animal species and humans for more than 50 years. A recurrent theme in all studies is progressive pulmonary ventilation, starting with the first breath [3,4,5,6]. However, the time required for complete pulmonary ventilation and the order in which different lung regions become ventilated remain unclear. Ventilation of all lung regions in neonatal calves was seen within 12 h using ultrasonography [12], 24 h using radiography [13], and 14 days after birth using computed tomography [14]. Pulmonary ventilation was reported to be progressive in all those studies, but whether certain lung regions become ventilated earlier than others, which remain nonventilated (silent spaces) or poorly ventilated, or whether all regions become ventilated more or less simultaneously was not determined. Electrical impedance changes can be measured in a breath cycle and used to assess changes in lung expansion and pulmonary air content. For instance, a large difference in impedance between the start and the end of inspiration in a certain lung region indicates more intense ventilation of that region compared with a region where impedance changes are smaller or non-existent. 

Of note, in our measurements only 9.8% of all lung regions were classified as silent spaces at the 15 min postnatal measurement, indicating that less than 10% of the lung is not or only minimally ventilated at that point, meaning than 90% of all alveoli are ventilated within minutes after birth. The presence and change over time of NSS and DSS have to be interpreted separately. In mechanically ventilated patients, NSS represent overdistended lung areas [30]. In our spontaneously breathing calves, the NSS can be more explained by the predefined lung area based on the finite element model (Figure 2C). As this model was reconstructed from CT scans of calves older than 1 week, it is very likely that the higher NSS directly after birth and decrease over time may be due to the fact that the thoracic cavity expands in the dorsal part of the thorax rather than truly nonventilated lung tissue.

However, the presence of DSS can be interpreted as nonventilated lung tissue as the anatomical shape of the thorax does not change extensively in the ventral parts over the first few weeks of life. DSS has also been used in several scenarios in preterm babies and lambs to identify nonventilated collapsed lung areas [38,39,40,41]. The magnitude of DSS we found in our calves is considerably higher than in humans [30]. The occurrence of high DSS in the calves can be attributed to an effect of gravity on extravascular lung water [30,42]. Considering the changes in DSS over the first hours of life, some assumptions can be made on the distribution and absorption of extravascular lung water, which represents mainly amniotic fluid in new-born calves. The first measurement at time 0 showed that all lung regions were for the most part ventilated, which means that the amniotic fluid was uniformly distributed. However, subsequent measurements showed a significant increase in DSS and dorsal displacement of the centre of ventilation within the first hour of life. This most likely reflected accumulation of fluid in ventral regions of the lungs because the calves were in sternal recumbency. Similar position-dependent changes in lung fluid distribution were also observed in term and preterm human babies [43] and other species with pulmonary oedema [16,44,45]. The rationale for the observed changes is that gravity causes accumulation of extravascular lung fluid in the dependent regions of the lungs. This causes a shift in distribution of ventilation towards the nondependent dorsal regions of the lungs, an effect that is described as the ‘slinky spring effect’, taking a vertically orientated spring that is fixed on top as a model. The spring is stretched in the top part and compressed at the bottom [46,47]. This dorsal shift in ventilation was also observed in the calves in this study represented by a significant increase in ventrodorsal CoV over the first hour of life. The shift of the centre from ventral to dorsal in the first hour of life was paralleled by an increase in DSS ventrally; a larger proportion of silent spaces in the ventral lung regions resulted in the centre being located more dorsally. This relationship was emphasized by a positive correlation between the two parameters.

After about 45 min, the percentage of DSS progressively declined, which was most likely due to absorption of amnionic fluid, similar to descriptions in neonatal rabbits and lambs [48,49]. This absorption of extravascular fluid began in rabbits 30 to 60 min after birth and then decreased continuously. Absorption of interstitial fluid is also believed to occur in neonatal foals and human babies and appears be complete by about 6 h after birth [50,51]. This time period corresponds with the most pronounced decrease in DSS seen in the present study. A recent radiographic study of pulmonary clearance in neonatal calves based on lung opacity showed that alveolar clearance is delayed when the duration of parturition increases [52].

The aforementioned ventrodorsal centre of ventilation was located dorsal to the theoretical centre of ventilation in all calves at all measurements, indicating greater ventilation of dorsal regions of the lungs. Likewise, in human babies, the CoV was at 55% to 60% and, thus, dorsal in the first 6 min after birth [40]. This is in concordance with a study evaluating the centre of ventilation in adult horses using the same EIT device as in the study described here where the CoV was >60% in a cohort of healthy horses [16]. No CoV information exists on other neonatal mammals. However, CT studies in calves showed greater ventilation in the dorsal regions of the lungs in the first 2 weeks of life, but ventilation of the ventral regions underwent greater increases than those dorsally [14]. 

Calculation of the CoV_rl_ and the local distribution of ventilation showed that the right lung had greater ventilation than the left lung throughout the study period and that approximately two-thirds of the inhaled air entered the right lung. The reasons for this are related to anatomical features. The right lung is considerably larger than the left lung in cattle and consists of four lobes compared with two lobes in the left lung. Furthermore, the right lung lobes are ventilated via two bronchi and the left lobes are ventilated via one [53], whereas the lung volume in the left hemithorax is smaller because of the heart. Measurements using EIT in new-born human babies also determined greater ventilation in the right lung [54,55]. The phenomenon that the right lung is preferentially ventilated also in adult mammals has been observed in humans, horses, and steers [16,56,57].

The division of the lung area into four regions (ventral, centroventral, centrodorsal, and dorsal) showed that the smallest proportion of ventilation occurred in the periphery of the ventral and dorsal areas of both lungs. Except for one time period, this did not change throughout the entire measuring period. Ventilation was considerably greater in the central regions compared with the peripheral regions, and, in the right lung, the centrodorsal part always had the greatest ventilation. This finding was in agreement with EIT studies in other mammals [16,56,57].

In the left lung, the centrodorsal region had the greatest ventilation only in the first 2 h after birth. During the 3 week study period, a significant increase in ventilation was seen in the centroventral region of the left lung, where the greatest ventilation was usually measured. The shift in ventilation to the more ventral region of the left lung after the absorption of extracellular lung fluid may have been related to the continuing decrease in airway resistance and the gradual increase in lung compliance, which increases in calves, especially in the first 24 h of life [58,59] but is generally lower in bovine species than in people because of greater segmentation of the lungs [60]. Furthermore, increased ventilation of the centroventral region of the left lung may have been due to anatomical reasons because the bulk of the left lung is relatively further ventral compared with the right lung [53].

The mean respiratory rate in the first 4 h ranged from 38 ± 11 to 50 ± 20 breaths/min, which was similar to reported rates in calves of 41 ± 7 to 59 ± 6 breaths/min in the first 6 h of life [59,61]. A significant decrease compared with the initial respiratory rate did not occur until day 11 even though a pronounced albeit not significant decrease happened in the first few hours of life. Partial pressure of arterial CO_2_, paO_2_, and pH are the main determinants of the respiratory rate, with paCO_2_ having the greatest effect in adults [62]. The paCO_2_ nadir and paO_2_ peak did not occur until 12 h, which was in agreement with the results of another study [7]. In human medicine, the response to increased paCO_2_ can be lower than expected in the first week of life [63]. As expected, the pH increased continually in parallel with the drop in paCO_2_, also in agreement with another report [7]. Only pH correlated with respiratory rate in the logistical regression. 

Decreasing respiratory rate was paralleled by an increase in ΔZVT taken as a surrogate for tidal volume, which has also been observed in the first hours of life in neonatal foals and human babies [64,65] and was reflected in a stable ΔZmin, which reflects a stable minute ventilation. Only at two time points was an increase in ΔZmin observed, which was due to a high respiratory rate during these two measurements. Furthermore, increasing lung compliance and decreasing airway resistance in the first hours of life combined with improved ventilation of various lung regions may have contributed to increased tidal volume. Body weight did not correlate with ΔZVT and, therefore, was not a likely cause of the increase, at least not in the first few days of life. Therefore, compared with the first measurement, the significant differences in ΔZVT from day 6 onward were most likely attributable to an increase in lung volume reflected also by the decrease in NSS and DSS. In young bulls, horses, and people, EIT and spirometry were used in combination, which allowed the calculation of the ventilation volumes with a high degree of accuracy [56,66,67]. We did not use spirometry and, therefore, could not determine absolute tidal volumes.

Of the blood gas parameters, paO_2_ correlated with ΔZVT, which was likely attributable to increased alveolar ventilation per breath and over time and, thus, improved gas exchange. This is also supported by the A–a gradient, which can be used to recognise gas exchange impairment due to low ventilation/perfusion (V/Q) match [68]. In healthy adult cattle, a normal A–a gradient is 10 to 15 mmHg while an average of 23.5 mmHg is considered normal in neonatal calves [7,69,70]. This value was reached in our calves 2 h after birth. However, a decrease of 10 mmHg occurred in these first 2 h, indicating a large improvement in gas exchange during this time period after birth. This drop in A–a gradient goes in parallel with a decrease in Fshunt volume. This may be due to the functional closure of the *foramen ovale* and the *ductus arteriosus* Botalli, which starts immediately after birth resulting in a gradual reduction of right-to-left shunting [71,72].

Our data did not show a direct correlation of the decrease in DSS, A–a gradient, and Fshunt, although a physiologic relationship is obvious. This lack in correlation is very likely due to a too low number of calves to show this specific correlation.

Another limitation arises from the fact that characterisation of ventilation of cranial and caudal lung regions was not possible because EIT captures only a lentiform region of the lung beneath the electrode belt according to the distribution of the electric current in the thorax [15]. Other limitations of the study included possible artefacts caused by movement of the calves, which may have affected measurements of several EIT variables, but comes with the nature of data collection in unsedated animals, which is preferable to study physiologic changes as the effect of sedatives on ventilation can be avoided. 

## 5. Conclusions

The results of our study show that some level of ventilation occurs in almost all regions of the lungs shortly after birth. Over time, nonventilated lung areas are recruited, and ventilation of already well-ventilated lung regions undergoes further improvement. EIT measurements suggest that the amniotic fluid is reabsorbed from the extravascular lung tissue within the first 2 h of life. The results obtained using EIT were also reflected by changes in blood gas variables. Our results may facilitate non-invasive assessment of functional or inflammatory lung disorders in calves in the future. 

## Figures and Tables

**Table 1:** Means and standard deviations of the blood gas variables pO_2_ (partial pressure of oxygen), pCO_2_ (partial pressure of carbon dioxide), and sO_2_ (oxygen saturation) in 20 neonatal calves in the first 24 h of life. Within rows, values with an asterisk differ significantly from the 15 min value. v, venous; a, arterial.

	0 min (v)	15 min (a)	2 h (a)	6 h (a)	12 h (a)	24 h (a)
pH	7.21 ± 0.08	7.29 ± 0.06	7.33 ± 0.04	7.34 ± 0.04 *	7.39 ± 0.03 *	7.41 ± 0.03 *
pO_2_ (mmHg)	26.77 ± 5.94	51.93 ± 12.54	62.87 ± 12.89	69.32 ± 10.58 *	74.01 ± 12.27 *	71.49 ± 12.50 *
pCO_2_ (mmHg)	67.53 ± 8.62	55.29 ± 4.01	54.36 ± 5.27	50.42 ± 6.61	45.33 ± 8.37 *	47.15 ± 4.47 *
sO_2_ (%)	50.70 ± 14.86	85.93 ± 7.71	93.05 ± 3.69 *	94.80 ± 2.79 *	95.67 ± 1.78 *	94.93 ± 2.09 *

## Data Availability

The data presented in this study are available on request from the corresponding author.
